# A Comparison of Web and Telephone Responses From a National HIV and AIDS Survey

**DOI:** 10.2196/publichealth.5184

**Published:** 2016-07-29

**Authors:** Marcella K Jones, Liviana Calzavara, Dan Allman, Catherine A Worthington, Mark Tyndall, James Iveniuk

**Affiliations:** ^1^ Faculty of Medicine University of Toronto Toronto, ON Canada; ^2^ The CIHR Social Research Centre in HIV Prevention Toronto, ON Canada; ^3^ Dalla Lana School of Public Health University of Toronto Toronto, ON Canada; ^4^ School of Public Health and Social Policy University of Victoria Victoria, BC Canada; ^5^ BC Centre for Disease Control Vancouver, BC Canada; ^6^ Faculty of Medicine University of British Columbia Vancouver, BC Canada

**Keywords:** survey methods, cross-sectional survey, community survey, HIV, AIDS, social desirability, data collection methods, data quality, health attitude, health knowledge, attitudes, practice

## Abstract

**Background:**

Response differences to survey questions are known to exist for different modes of questionnaire completion. Previous research has shown that response differences by mode are larger for sensitive and complicated questions. However, it is unknown what effect completion mode may have on HIV and AIDS survey research, which addresses particularly sensitive and stigmatized health issues.

**Objectives:**

We seek to compare responses between self-selected Web and telephone respondents in terms of social desirability and item nonresponse in a national HIV and AIDS survey.

**Methods:**

A survey of 2085 people in Canada aged 18 years and older was conducted to explore public knowledge, attitudes, and behaviors around HIV and AIDS in May 2011. Participants were recruited using random-digit dialing and could select to be interviewed on the telephone or self-complete through the Internet. For this paper, 15 questions considered to be either sensitive, stigma-related, or less-sensitive in nature were assessed to estimate associations between responses and mode of completion. Multivariate regression analyses were conducted for questions with significant (*P*≤.05) bivariate differences in responses to adjust for sociodemographic factors. As survey mode was not randomly assigned, we created a propensity score variable and included it in our multivariate models to control for mode selection bias.

**Results:**

A total of 81% of participants completed the questionnaire through the Internet, and 19% completed by telephone. Telephone respondents were older, reported less education, had lower incomes, and were more likely from the province of Quebec. Overall, 2 of 13 questions assessed for social desirability and 3 of 15 questions assessed for item nonresponse were significantly associated with choice of mode in the multivariate analysis. For social desirability, Web respondents were more likely than telephone respondents to report more than 1 sexual partner in the past year (fully adjusted odds ratio (OR)=3.65, 95% CI 1.80-7.42) and more likely to have donated to charity in the past year (OR=1.63, 95% CI 1.15-2.29). For item nonresponse, Web respondents were more likely than telephone respondents to have a missing or “don’t know” response when asked about: the disease they were most concerned about (OR=3.02, 95% CI 1.67-5.47); if they had ever been tested for HIV (OR=8.04, 95% CI 2.46-26.31); and when rating their level of comfort with shopping at grocery store if the owner was known to have HIV or AIDS (OR=3.11, 95% CI 1.47-6.63).

**Conclusion:**

Sociodemographic differences existed between Web and telephone respondents, but for 23 of 28 questions considered in our analysis, there were no significant differences in responses by mode. For surveys with very sensitive health content, such as HIV and AIDS, Web administration may be subject to less social desirability bias but may also have greater item nonresponse for certain questions.

## Introduction

Choosing a mode of data collection is one of the most important decisions that researchers make when designing a research survey. The choice of mode may be shaped by feasibility, cost, response rates, and potential for achieving a more representative sample. Currently, two of the most frequently used administration modes are telephone and Web [1,2]; the former is often interviewer-administered and the latter is usually self-administered. Often, telephone questionnaires have higher response rates and can also allow interviewers to clarify questions and motivate participants. However, telephone questionnaires can be relatively resource intensive and may have difficulty using visual aids [3,4]. By contrast, Web questionnaires are often less expensive and easier to implement but may be affected by lower response rates, item nonresponse, greater potential for fraud, and Internet accessibility issues that affect representativeness [3,5-7]. Given the compromises between modes, another option is a mixed-mode survey, which is administered using two or more data collection modes to allow participants an element of convenience and choice and to also compensate for the limitations of a single mode [1,2,8-10]. In each case, the choice of mode, or modes, may influence respondents’ answers, and accordingly, the conclusions that researchers can draw about a study population.

One recurring concern in the literature is the relationship between survey mode and the accuracy of the information provided by the respondent because survey mode may influence respondents’ willingness to answer certain questions at all, truthfully, or with socially desirable but inaccurate answers. Social desirability bias may be heightened in interviewer-administered telephone and face-to-face surveys but reduced in self-administered Web surveys [11,12]. Furthermore, although Web surveys typically have lower response rates than other modes of survey research, they can have lower item nonresponse rates as well, possibly because of the absence of interviewers whose characteristics can influence the kinds of attitudes and behaviors that people report [12-14]. In the context of mixed-mode surveys, these challenges can lead to inaccurate conclusions if researchers aggregate data that were collected through different modes [10], or if they compare results across multiple surveys that were collected by different modes [12]. In addition, if respondents are given the choice of mode, respondents may select a mode where the provision of socially desirable answers is more prevalent, or choose to not respond to certain questions, which may also prevent researchers from making accurate comparisons across subgroups [15,16].

The emergence of Web surveys has provided researchers with technology that creates new opportunities and challenges for addressing response bias. Accordingly, there has been much recent research on Web surveys and how mode effects may shape responses to survey questions [5,9,10,17-23]. Although most research shows that social desirability bias is lower in Web surveys compared with other modes [4,12,24], other work has shown no difference [25,26]. This suggests that Web surveys may reduce social desirability bias only under certain circumstances and potentially only with certain types of questions. Alternatively, as Internet use is increasingly ubiquitous in society, the literature may be starting to reflect the fact that Web surveys may no longer evoke differential responses from survey participants.

In particular, how willing respondents are to report personal, private, or sensitive matters in a survey setting can affect data quality [9,17,18,22,27,28]. In a recent meta-analysis of 10 experimental studies that looked at Web administration versus interviewer-administered modes for collecting potentially embarrassing information, the authors concluded that self-administration via the Web improved reporting accuracy for socially undesirable responses [18]. Similarly, recent findings from the British National Survey of Sexual Attitudes and Lifestyles compared computer-assisted personal interviews with self-interview modes and found that reporting of sensitive information was overall higher in the Web/self-administered survey mode [21]. This could make Web surveys the preferred mode for asking highly sensitive personal questions.

This body of research is encouraging for research fields that investigate potentially sensitive topics. HIV and AIDS–related research often addresses particularly sensitive issues, and avoiding sensitive questions is not possible in most HIV- or AIDS-related surveys. Given the sensitive content, it is likely that studies of HIV and AIDS face particularly strong challenges arising from reporting biases, including social desirability and item nonresponse biases. To our knowledge, no national population-based experimental studies have looked at response differences between telephone and Web questionnaires for extremely sensitive health content, such as HIV and AIDS. Therefore, in this area of health research, observational studies that do not randomize participants to a completion mode can provide a helpful foundation for which to further explore the issue of response bias by mode. Indeed, it may be advantageous for health researchers considering mixed-mode survey designs to learn whether their specific topic is prone to response bias by mode, even when that data are nonexperimental in nature, such as 2 recent observational studies that explored response biases for specific health issues [26,27]. Understanding the extent to which responses may differ and how they differ depending on mode is essential given the use of mixed-mode designs and for the comparability of studies on sensitive health topics that use different data collection modes.

A national HIV and AIDS survey was conducted among Canadians in 2011 to determine public knowledge, attitudes, and behaviors about HIV and AIDS. The survey used a mixed-mode data collection method, whereby participants selected whether they would prefer to complete the questionnaire by telephone or the Internet, which allows us to compare responses between (persons who chose) telephone and Web completion modes. We seek to determine whether the mode of questionnaire completion influences responses in terms of social desirability and missing data. To explore whether any observed associations vary by question type, we select different types of questions including potentially sensitive, stigma-related, and less-sensitive questions.

## Methods

### Questionnaire Development

The bilingual questionnaire was developed based on both literature reviews and the expertise of researchers and other professionals at the Canadian Institutes of Health Research (CIHR) Social Research Centre in HIV Prevention and the Canadian Foundation for AIDS Research. Questions were developed to resemble previous large-scale national HIV surveys for comparative purposes [29]. The final questionnaire contained sociodemographic items and questions about HIV and AIDS knowledge, attitudes, and behaviors (for more details, see [30]). Due to the study's sensitive nature, the survey was pretested among a sample (n=100) and monitored for issues (none were identified). Ethics approval was obtained from the University of Toronto Ethics Review Board.

### Survey Administration

The survey was conducted in English and French by The Strategic Counsel, a polling and market research firm, between May 5 and May 25, 2011 among individuals aged 16 years and older in all Canadian provinces and territories. Participants were selected using a 2-stage sampling design. In the first stage, participants were randomly sampled from the general population using a random-digit-dial that used both cellular and landline telephone numbers. Calls were managed by an interactive voice response system, with numbers retired from the system after 1 initial call and 3 unanswered callbacks. Once contacted, individuals entered basic sociodemographic information (age, gender, postal code) on their telephone keypads and were asked to participate in a survey at a later date. If the participant agreed, they were added to a panel of willing participants. For the second sampling stage, these panel participants comprised the sampling frame and were sampled directly (with stratification by region) and were contacted by a live interviewer who introduced the panel’s incentive scheme and invited participants to complete the questionnaire over the Internet or by telephone. The incentive scheme was used to exclude professional respondents and awarded participants with charity dollars (eg, option to donate to a charity of choice) and a ticket for a monthly raffle prize. The survey methodology is also described elsewhere [30-32]. The blended participation rate for the survey was 25%, with participation higher among those who completed the survey by telephone compared with those who did so through the Internet (31.1% and 18.4%, respectively). For this analysis, we exclude 16-17-year olds (n=54) because of their low Web-based completion (n=2).

### Measures

A total of 15 questions were selected from the 85 questions on the questionnaire to test differences in responses and were categorized by question type (Table 1). This subset of questions was used to minimize the effect of multiple comparisons testing. We chose 5 sensitive and 5 stigma-related questions that we hypothesized might be affected by response biases. We also chose 5 relatively benign or less-sensitive questions as controls. We use the terms “sensitive,” “stigma-related,” and “less-sensitive” as descriptors for the question types to organize our approach and interpretation; however, study participants were not aware of these categories and will have uniquely interpreted the sensitivity of each question.

As outlined in Table 1, to study differences in social desirability, we tested 13 questions that were suitable for predicting a socially desirable response. Two of the questions were excluded from this analysis because we did not believe there was a “socially correct” response, and if a mode difference were to be found, it would be difficult to interpret the directionality of the difference with respect to social desirability bias. Most questions had binary (yes or no) or Likert scale responses, except for question 1 (HIV or AIDS testing), where answers were recoded as voluntary versus nonvoluntary/nontested (using the same definition as in [32]), and for question 3 (number of sexual partners), where answers were recoded as 1 versus greater than 1. To study differences in missing data (eg, item nonresponse), all 15 questions were used. For analytical purposes, responses were dichotomized: missing or not missing. Both the “do not know/not sure” and “prefer not to answer” responses were grouped with missing responses because they were coded as missing during data entry. Complete details about the questions, including the response keys, are available in the Web-based Multimedia Appendix 1.

Our independent variable of interest, mode of completion, is a binary variable generated from each respondent’s mode of questionnaire completion—web or telephone. Additional independent variables were self-reported sociodemographic items; these included age, gender, highest level of education attained, household income, sexual minority status, visible minority status, and Canadian region, all of which were treated as categorical variables in the analysis. Minority status was defined by the respondent indicating that they belonged to a “visible minority” group or a “sexual minority” group.

**Table 1:** An overview of the questions selected for analysis by question type and study objective (complete details about the questions are available in Web-based Multimedia Appendix 1).

Question type^a^	#	Question	Objective #1	Objective #2
			Social desirability	Item nonresponse
**Sensitive**				
	1	Have you ever been tested for HIV or AIDS for any of the following reasons?	✓	✓
	2	Have you ever had sex in your lifetime?	✓	✓
	3	How many different partners have you had sexual intercourse with in the last 12 months?^b^	✓	✓
	4	Were any of these casual partners? In other words, were they someone that you are not in a regular or long-term relationship?^b^	✓	✓
	5	What is your annual household income from all sources before taxes?^c^	-	✓
**Stigma-related**				
	6	I could not become friends with someone who has HIV or AIDS (select level of agreement with statement)	✓	✓
	7	I feel afraid of people living with HIV or AIDS (select level of agreement with statement)	✓	✓
	8	People living with HIV or AIDS have the right to be sexually active (select level of agreement with statement)	✓	✓
	9	How comfortable or uncomfortable would you be with a close friend or family member dating someone with HIV or AIDS? (select level of comfort)	✓	✓
	10	How comfortable or uncomfortable would you be with shopping at a small neighborhood grocery store, if you found out that the owner had HIV or AIDS? (select level of comfort)	✓	✓
**Less-sensitive**				
	11	Thinking about illnesses or diseases, what is the one illness or disease that concerns you the most? (open ended)^c^	-	✓
	12	In the past year, did you actively seek out or look for information about HIV or AIDS?	✓	✓
	13	Do you recall donating to any charitable or not-for-profit organization in the last year?	✓	✓
	14	To what extent do you believe it is government’s responsibility to continue to fund HIV or AIDS research?	✓	✓
	15	How knowledgeable would you say that you are about HIV or AIDS?	✓	✓
		Total	13	15

^a^Question types are categories that are used to guide our analysis and may not be perceived this way by participants.

^b^Were only asked to those who were sexually active in the past 12 months.

^c^Were excluded from objective 1 because we did not believe there was a “socially correct” response.

### Statistical Analyses

Analyses were performed with Stata IC v. 12 using its survey data analysis program. All reported results are weighted to represent the Canadian population in 2011 in terms of age, gender, and province or territory of residence. Standard errors were estimated using linearized or robust variance estimators, and 95% CIs are presented where appropriate. Descriptive statistics and bivariate associations with mode of completion were generated for all sociodemographic variables. Bivariate associations between mode of completion and the selected questions were generated using Pearson’s chi-square or Wald tests as appropriate.

Any significant bivariate associations observed were either deemed attributable to sample differences in the study population between the telephone and Web groups or to the completion mode itself. To address the first possibility, sample differences between mode groups, a multivariate analysis was conducted. Multivariate regression analyses were conducted for only those questions with significant differences (*P* ≤.05) in responses or missing responses between telephone and Web administration modes. Separate regressions were run for each question: logistic regression was used for binary responses and linear regression for continuous responses. First, sociodemographic variables with significant bivariate associations (*P* ≤.05) with mode of administration were included in the regression models as control variables. Second, to minimize the effect of mode selection bias on confounding our results, we use propensity score methodology, which was developed to approximate the analysis of observational (nonrandomized) data to that of randomized treatment assignment [33]. The propensity score balances systematic differences between the telephone and Web response groups so that observed sociodemographic covariates are similar between the 2 groups [33]. Therefore, the inclusion of the propensity score as a covariate in our multivariate analysis helps reduce bias that may be present as a result of respondents’ self-selection into telephone or Web response modes. We generated a propensity score using a logistic regression model in which mode of completion was regressed on all the observed sociodemographic characteristics in our study (age, gender, education, income, sexual minority, ethnic minority, and province of residence). An individual’s estimated propensity score is therefore the predicted probability of that individual choosing to complete their survey on the Internet. After the propensity score variable was generated, it was added as a covariate in our multivariate models, by simply including it as an independent variable in the model statement.

Respondents with any missing observations for covariates were excluded from the regression analysis, with the exception of missing household income values where an additional response category was generated to maintain sample size. All logistic regression models satisfied the Hosmer–Lemeshow test for goodness of fit (*F*-adjusted mean residual >.05) [34].

## Results

### Characteristics of the Study Population

In total, 2085 Canadians aged 18 years and older completed the questionnaire. Overall, the unweighted sample closely reflected the actual distribution of the 2011 Canadian population in terms of key demographic variables such as age, gender, and province or territory (Table 2). The study sample is more highly educated, however, than the general Canadian population. A total of 1690 participants (81.0%) completed the questionnaire through the Internet, and 395 (19.0%) completed by telephone. Table 3 presents the sociodemographic characteristics of the study sample by mode of questionnaire completion. Mode was significantly associated with most of the sociodemographic characteristics we considered, except for visible minority status. Compared with Web respondents, telephone respondents tended to be older (*P*<.001), were more likely to be female (*P*=.05), reported less education (*P*<.001), had lower household incomes (*P*<.001), and were more likely to live in the province of Quebec (*P*<.001). Those who self-identified as being a member of a sexual minority group were more likely to have chosen to complete the questionnaire through the Internet (*P*=.004).

### Objective 1: Responses to Questions According to Mode of Completion

Responses for the 13 social desirability questions overall and by mode of questionnaire completion are presented in Table 4. Significant differences in responses were observed for 5 of the 13 questions. Among those who had been sexually active in the last 12 months, a significantly higher proportion of Web respondents (13.8%) than telephone respondents (4.3%) reported having more than 1 sexual partner in the last 12 months (*P*<.001). Web respondents reported a higher level of comfort with shopping at a small neighborhood grocery store where the owner was known to have HIV or AIDS (3.20 vs 2.92 for telephone respondents, *P*<.001 *).* In response to whether survey participants agreed with the stigma-related statement “I feel afraid of people living with HIV and AIDS,” the overall mean level of agreement in the study population was low (2.53), which falls between “2—disagree” and “3—somewhat disagree.” Web respondents tended toward “disagree” (2.48), whereas telephone respondents tended toward “somewhat disagree” (2.74; *P*=.021). Web respondents also reported more charitable giving in the past year (86.5%) compared with telephone respondents (77.4%; *P*<.001) and greater self-reported knowledge of HIV or AIDS (4.56 vs 4.39 for telephone respondents, *P*=.046). The remaining 8 questions showed no statistically significant differences in responses between the Web and telephone respondents.

### Objective 2: Missing Data According to Mode of Completion

Missing responses for the 15 questions overall and by mode of completion are summarized in Table 5. Overall, the frequency of missing data was low and ranged from 0.9% to 4.7% with the exception of 2 questions: annual household income (15.2% missing) and illness or disease that concerns you the most (9.8% missing). Mode did not affect refusing to report annual household income. Significant differences in missing responses were observed for 3 of the 12 questions. A missing or do not know response to whether the respondent had tested for HIV was significantly more likely in Web respondents (4.9%) than telephone respondents (0.7%; *P*<.001). Web respondents were also more likely to have a missing or do not know response when asked to rate their comfort level with shopping at a grocery store owned by someone who has HIV or AIDS (5.2% vs 2.7% for telephone respondents, *P*=.041) and when asked about what illness or disease concerns them the most (11.1% vs 4.3% for telephone respondents, *P*<.001). No significant differences in missing responses between the modes were observed for the remaining 12 questions.

**Table 2:** Key variables from the 2011 general population in Canada, aged 18 years and older, compared with unweighted and weighted survey samples (n=2085).

Variable	Categories	Canadian Population^a^ %	Unweighted survey sample %	Weighted survey sample %
Age				
	18-24	11.5	10.1	12.1
	25-39	24.4	26.7	25.0
	40-49	18.8	16.9	16.8
	50-59	18.8	19.5	19.2
	60+	26.3	26.9	26.9
Gender				
	Male	48.5	49.3	48.2
	Female	51.5	50.7	51.8
Education^b^				
	Male	48.5	49.3	48.2
	Female	51.5	50.7	51.8
	University	16.5	28.0	28.0
	Grad or postgrad	9.4	17.3	17.2
Region				
	British Columbia	13.4	13.0	12.3
	ALberta	10.6	10.1	10.2
	Saskatchewan	3.0	3.7	3.0
	Manitoba	3.5	3.9	3.5
	Ontario	38.3	36.8	38.3
	Quebec	23.9	23.5	24.0
	New Brunswick	2.3	2.0	2.3
	Nova Scotia	2.8	4.1	3.2
	Prince Edward Island	0.4	0.4	0.3
	Newfoundland and Labrador	1.6	1.3	1.6
	Yukon	0.1	0.8	0.8
	Northwest	0.1	0.4	0.4
	Nunavut	0.1	0.1	0.1

^a^Based on 2011 census of Canada [35-37].

^b^Education shown for age 25 years and older (n=1860), as the Canadian Census does not report highest level of education for less than 25 years.

**Table 3:** Sociodemographics of study population by mode of questionnaire completion.

Variable	Categories	Overall^a^ % (n=2085)	Web % (n=1690)	Telephone % (n=395)	*P* value^b^
Overall (n=2085)		100	81.0	19.0	
Age (n=2085)					
	18-24	12.1	13.1	7.9	<.001
	25-39	25.0	28.0	12.3	
	40-49	16.8	17.1	15.4	
	50-59	19.2	18.7	21.5	
	60+	26.9	23.1	42.9	
Gender (n=2085)					
	Male	48.2	49.3	43.8	.050
	Female	51.8	50.7	56.2	
Education (n=2069)					
	≤High school	20.8	14.3	48.3	<.001
	College	31.9	32.5	29.3	
	University	31.9	35.4	17.2	
	Grad or postgrad	15.5	17.8	5.3	
Income (n=2085)					
	<$40,000	23.3	19.6	39.1	<.001
	$40,000-$80,000	28.4	28.1	29.6	
	>$80,000	33.2	37.8	13.7	
	Prefer not to answer	15.2	14.6	17.7	
Sexual minority (n=2038)		4.8	5.5	2.0	.004
					
Visible minority (n=2038)		7.7	7.5	8.6	.462
					
Region (n=2085)					
	West	29.1	30.6	22.7	<.001
	Ontario	38.3	39.1	35.0	
	Quebec	24.0	21.6	34.1	
	East	7.4	7.3	8.1	
	Territories	1.3	1.5	0.2	

^a^Overall % based on “n” from cross tabulation.

^b^Pearson chi-square corrected for weighted data (design-based F).

**Table 4:** Objective 1 (social desirability): responses to questions by mode of questionnaire completion.

Question type	Question	Response or scale^a^	Overall^b^ % or mean (standard error-SE)	Web % or mean (SE)	Telephone % or mean (SE)	*P* value^c^
Sensitive questions						
	Voluntary testing for HIV or AIDS (n=1997)	Yes	29.7	29.9	29.0	.739
	Sexual activity in lifetime (n=2054)	Yes	96.7	96.6	96.9	.735
	More than 1 sexual partner in last 12 months (n=1455)^d^	Yes	12.4	13.8	4.3	<.001
	Casual sex partners in last 12 months (n=1461)^d^	Yes	12.2	12.8	8.7	.089
	Could not become friends with someone who has HIV or AIDS (n=2055)	1-7	1.93 (0.04)	1.91 (0.04)	2.04 (0.09)	.169
	Afraid of people living with HIV or AIDS (n=2065)	1-7	2.53 (0.04)	2.48 (0.04)	2.74 (0.11)	.021
	People with HIV or AIDS have the right to be sexually active (n=2005)	1-7	4.39 (0.05)	4.35 (0.05)	4.57 (0.11)	.084
	Comfort with a close friend or family member dating someone with HIV or AIDS (n=2008)	1-4	2.37 (0.02)	2.37 (0.03)	2.38 (0.05)	.897
	Comfort with shopping at small grocery store owned by someone who has HIV or AIDS (n=1987)	1-4	3.15 (0.02)	3.20 (0.02)	2.92 (0.06)	<.001
Less-sensitive questions						
	Actively seek information about HIV or AIDS (n=2061)	Yes	8.3	8.4	8.2	.919
	Charitable giving in the past year (n=2049)	Yes	84.7	86.5	77.4	<.001
	Government responsibility to fund HIV or AIDS research (n=2053)	1-7	5.54 (0.04)	5.51 (0.04)	5.65 (0.09)	.136
	Perceived HIV knowledge (n=2080)	1-7	4.52 (0.03)	4.56 (0.03)	4.39 (0.08)	.046

^a^1-7 Likert scale; 1=completely disagree, 4=neither agree nor disagree, and 7=completely agree. 1-4 Likert scale; 1=very uncomfortable, and 4=very comfortable.

^b^Overall % or mean based on “n” from cross tabulation.

^c^Pearson chi-square (categorical variables) or Wald test (continuous variables) corrected for weighted data (design-based F).

^d^These questions were only asked to those who were sexually active in the past 12 months.

**Table 5:** Objective 2 (item nonresponse): missing or do not know^a^ responses for questions by mode of questionnaire completion (n=2085, unless otherwise indicated).

Question type	Question	Overall^b^ %	Web %	Telephone %	*P* value^c^
Sensitive questions					
	Tested for HIV or AIDS	4.1	4.9	0.7	<.001
	Sexual activity in lifetime	3.4	3.6	2.3	.212
	Number of sexual partners in last 12 months^d^	1.3	1.4	0.8	.476
	Casual sex partners in last 12 months^d^	0.9	1.1	0.0	.127
	Annual household income	15.2	14.6	17.7	.129
Stigma-related questions					
	Could not become friends with someone who has HIV or AIDS	1.4	1.7	0.5	.082
	Afraid of people living with HIV or AIDS	1.0	0.8	1.8	.061
	People with HIV or AIDS have the right to be sexually active	3.8	3.7	4.3	.567
	Comfort with a close friend or family member dating someone with HIV or AIDS	3.4	3.9	3.4	.678
	Comfort with shopping at small grocery store owned by someone who has HIV or AIDS	4.7	5.2	2.7	.041
Less-sensitive questions					
	Illness or disease that concerns you the most	9.8	11.1	4.3	<.001
	Actively seek information about HIV or AIDS	1.1	1.3	0.3	.085
	Charitable giving in the past year	1.7	1.9	1.0	.249
	Government responsibility to fund HIV or AIDS research	1.5	1.6	1.0	.406
	Perceived HIV knowledge	0.2	0.2	0.3	.983

^a^All responses recategorized into binary variables: missing or do not know (yes) or other (no).

^b^Overall % based on “n” from cross tabulation.

^c^Pearson chi-square corrected for weighted data (design-based F).

^d^These questions were only asked to those who were sexually active in the past 12 months (n=1474).

### Findings from the Multivariate Analysis

The 8 questions that exhibited significant associations with mode in the bivariate analysis were entered into separate regression models to control for potential confounding by differences between the online and telephone samples. Adjusted associations with mode of completion and these questions are shown in Table 6. After adjusting for sociodemographic differences between the Web and telephone groups, differences in responses remain for 5 of the 8 questions. When the propensity score is added as a covariate to adjust for mode selection bias, the significant differences remain. While the propensity score does not resolve unmeasured differences between the two groups, the fully adjusted estimates are controlled for any confounding due to observed systematic differences that predict choice of response mode. Web respondents had 3.65 greater odds of reporting more than one sexual partner in the last 12 months, compared to telephone respondents (sociodemographic and propensity score adjusted odds ratio (OR)=3.65, 95% CI 1.80-7.42). Those who completed online were also more likely than telephone respondents to report charitable giving in the past year (OR=1.63, 95% CI 1.15-2.29). In terms of item non-response, web respondents were significantly more likely to have a missing or “don’t know” response to questions about HIV testing (OR=8.04, 95% CI 2.46-26.31), comfort with shopping at small grocery store owned by someone who has HIV or AIDS (OR=3.11, 95% CI 1.47-6.63), and most concerning illness or disease (OR=3.02, 95% CI 1.67-5.47). After multivariate adjustment, the previously observed significant differences in responses between the modes for the other three questions did not persist. This suggests that the bivariate associations for these questions can be attributed to sociodemographic differences between the samples.

**Table 6:** Adjusted associations^a^ between mode of questionnaire completion (exposure) and 8 selected questions (outcomes; see Multimedia Appendix 2 on the Internet for full regression results).

Question type and question		Mode	Sociodemographic adjusted^b^		Sociodemographic^b^ and propensity score adjusted	
			Odds ratio or β coefficient	95% CI	Odds ratio or β coefficient	95% CI
**Objective 1: social desirability**						
	Sensitive: More than 1 sexual partner in the last 12 months (n=1424)					
		Telephone	1.00	—	1.00	—
		Web	3.76^d^	(1.86-7.59)	3.65^d^	(1.80-7.42)
	Stigma-related: afraid of people living with HIV or AIDS (n=2008)^c^					
		Telephone	0.00	—	0.00	—
		Web	0.000	(−0.23-0.23)	0.019	(−0.21-0.25)
	Stigma-related: comfort with shopping at small grocery store owned by someone who has HIV or AIDS (n=1934)^c^					
		Telephone	0.00	—	0.00	—
		Web	0.087	(−0.34-0.21)	0.069	(−0.05-0.19)
	Less-sensitive: Charitable giving in the past year (n=1996)					
		Telephone	1.00	—	1.00	—
		Web	1.61^d^	(1.15-2.27)	1.63^d^	(1.15-2.29)
	Less-sensitive: perceived HIV knowledge (n=2022)^c^					
		Telephone	0.00	—	0.00	—
		Web	−0.049	(−0.22-0.12)	−0.057	(−0.23-0.11)
**Objective 2: Item Non-Response**					
	Sensitive: tested for HIV or AIDS (n=2027)					
		Telephone	1.00	—	1.00	—
		Web	8.68^d^	(2.63-28.67)	8.04^d^	(2.46-26.31)
	Stigma-related: comfort with shopping at small grocery store owned by someone who has HIV or AIDS (n=2027)					
		Telephone	0.00	—	0.00	—
		Web	2.99^d^	(1.41-6.32)	3.11^d^	(1.47-6.63)
	Less-sensitive: illness or disease that concerns you the most (n=2027)					
		Telephone	1.00	—	1.00	—
		Web	3.01^d^	(1.68-5.38)	3.02^d^	(1.67-5.47)

^a^Separate logistic regression analyses were run for all questions except for continuous outcomes.

^b^Adjusted for age, gender, education, household income, sexual minority status, and region.

^c^Linear regression analysis.

^d^Indicates statistical significance at the 95% confidence level.

## Discussion

We sought to determine whether there are differences in social desirability and missing data between people who chose to complete an HIV- and AIDS-related questionnaire by telephone versus the Web. We anticipated that due to the sensitive and personal nature of some of the questions, we would see differences in responses between the telephone interviewer-administered questionnaire and the Web-based self-completed questionnaire. Although we saw large sociodemographic differences between completion modes, for most of the questions (23 of 28) we studied, there were no significant differences in responses by mode. Overall, 2 of 13 questions assessed for social desirability, and 3 of 15 questions assessed for item nonresponse were significantly associated with choice of mode in the multivariate analysis. However, despite finding few significant response differences, those we found were large in magnitude; ranging from 1.6- to 8-fold difference.

Participants were given a choice to complete the questionnaire through the Internet or by telephone. As expected, and similar to other mixed-mode studies that used a non-random method for selecting participants [17,27,38], we see large differences in sociodemographic characteristics between the Web and telephone groups. The telephone group was older and had a larger proportion of women. Similar to other Canadian studies, we also found that Internet respondents reported higher incomes and more education [27,38]. Other studies have found younger age, higher incomes, greater education, and race to be strongly associated with the Internet and email access [6,39-41]. Although we do not know whether that telephone respondents in our study have access to the Internet, our results are consistent with what these findings suggest about Internet access. The exception is that we found no difference in the proportion of visible minorities between the two modes; this finding may be unique to the Canadian population as compared with the American population, owing to more targeted social policies in Canada to expand Internet access and reduce the digital divide for disadvantaged and racialized groups [42].

We thought that sensitive and stigma-related questions might be differentially affected by social desirability between completion modes, but a pattern in responses by question type was not apparent. We saw some evidence of social desirability for sensitive questions, with telephone respondents reporting lower numbers to questions about sexual partners they had in the past year and in answering whether any of these sexual partners were casual partners. This is in line with previous research that has found that Web questionnaires are better at eliciting truthful responses to sensitive questions than telephone questionnaires [9,20,22,27,28]. However, only the question about number of sexual partners showed a statistically significant difference, a difference that remained after multivariate adjustment. This suggests that the social interaction with the interviewer may have resulted in respondents stating a lower number of sexual partners—a more socially appropriate response.

Four other questions that we tested for social desirability showed significant differences in responses by mode, but not in the anticipated direction. If social desirability was at play, telephone respondents would be expected to provide more tolerant responses about their attitudes toward people living with HIV and AIDS because these attitudes are more socially appropriate. However, we found that telephone respondents gave less tolerant responses than Web respondents when asked if they felt afraid of people living with HIV and AIDS and when rating their comfort level with shopping at a grocery store owned by someone who is HIV seropositive. Yet, these differences did not remain in the multivariate analysis and are therefore attributed to sample differences between mode groups. Similarly, telephone respondents reported less knowledge about HIV and AIDS and less charitable giving than Web respondents, which are considered socially undesirable responses, yet only, the difference in charitable giving persisted in the multivariate analysis. This result suggests that when reporting their charitable donations to a live interviewer, telephone respondents were not affected by social desirability and did not feel socially obligated to appear more charitable. Although we adjusted for income and education in our models, Web respondents may be different from telephone respondents in other important characteristics that could account for the counterintuitive result to this question, such as religious affiliation, awareness of need, altruism, and personal values, all of which are known motivators for charitable giving [43]. Another possible explanation is that some telephone respondents may fear that the live interviewer will ask them to donate to a charity at the end of the questionnaire if they appear charitable and therefore choose to answer “no” to avoid the perceived solicitation.

With respect to the missing data objective, we also saw no clear pattern of response differences between question types. Among the questions we considered, there was generally a greater frequency of item nonresponse to sensitive and stigmatizing questions and among the Web sample. One question from each category was found to have significant differences in item nonresponse between the telephone and Web groups, with a greater frequency of missing responses in the Web questionnaire. These differences also remained in the multivariate analysis, which suggests that they are attributable to the mode and not to sociodemographic differences between the samples. The greatest amount of item nonresponse was for annual household income (15.2%), with more nonresponse among telephone participants (17.7%) than Web participants (14.6%), although this difference was not statistically significant. Questions about income are well known to generate a large frequency of missing data in most surveys.

Our findings for item nonresponse are consistent with those of other experimental and observational studies that looked at missing data by mode and also found that Web questionnaires produced more missing data [9,27,44,45]. It is thought that telephone interviewers may inadvertently persuade respondents to answer, or that they may further explain or clarify questions. Some of these studies found that differences were particularly evident for complicated or difficult questions [9], although neither question that we saw differences for is considered difficult. Due to the nature of how the data were entered and coded, we are unable to distinguish between different types of missing data (eg, “do not know” responses vs not answered questions), and therefore, we cannot tell where “do not know” responses may be genuine answers. The concerning illness or disease question was an open-ended question, and this likely contributed to item nonresponse among the Web sample as typing was required, and no interviewer was present to prompt for a response. This is consistent with other studies that have also found increased missing data for open-ended questions on Web surveys [22,27]. In contrast, the HIV testing question provided response categories, but some respondents may not have known if they had ever been tested for HIV and legitimately selected the “do not know” response, which would have contributed to item nonresponse for this question.

Our study benefits from several strengths. We conducted a large national survey, and so, we anticipate that our results have fair generalizability to the Canadian population. Our sample was recruited from a respondent panel that was constructed using random-digit-dial, and this strengthens our ability to make causal inferences [46]. We also performed a multivariate regression analysis to control for sample differences between the telephone and Web groups and included a propensity score that can help adjust for mode selection effects [10]. After accounting for sociodemographic disparities between the groups, it is more likely that any remaining differences in responses can be attributed to the mode of completion.

The results of our observational study should be considered along with its limitations. First, and most importantly, we did not randomize respondents to mode, and therefore, our estimates may be subject to selection bias. We attempted to control for the risk of selection bias by including a propensity score in our regression models. The inclusion of the propensity score neither changed the significance of our results from the sociodemographic-adjusted models nor did it greatly change their magnitude. This indicates that relatively little selection bias is present. Furthermore, the fact that our results persist in the face of the propensity score control would suggest that our results do describe the effect of survey mode on response patterns. However, there may be other unmeasured characteristics that we are unable to control for, such as differences in people’s experiences and opinions between the groups, and if these characteristics also affect mode selection, this could be confounding our results. Although, if these characteristics are unrelated to mode selection, they would only produce nondifferential misclassification, which would indicate our estimates are conservative. Furthermore, the regression model that generated the propensity score was limited to variables contained within our questionnaire, and as such, there may be variables that help explain why one chose a given survey mode that are missing from the model specification. The propensity score is therefore not a perfect predictor of mode selection, and there is some degree of misspecification as the probability of choosing a mode and actually choosing it are distinct constructs. In addition, because the sample size of telephone respondents was much smaller than the sample of Web respondents, the propensity score may not be able to adequately balance sociodemographic variables across the response groups. Second, the participation rate from the random-digit-dial panel was low, and more people who opted to complete by telephone finished the questionnaire (31.1%) than those who opted to complete through the Internet (18.4%). Although this participation rate limits the external validity of our results, particularly for the Web sample, it is nevertheless consistent with response rates from similar surveys in Canada that report response rates between 14% and 28% [31,47]. Finally, beyond social desirability and missing data, we did not consider other potential ways that responses could differ between modes, such as nondifferentiation bias (eg, answering the same response across a series of items), acquiescence bias (eg, agreeing across questions), and avidity bias (eg, disproportionate representation of those interested or invested in the survey topic of HIV and AIDS), due to study scope and design of the questionnaire.

In summary, there has been considerable concern in the survey methodology literature about the use of Web questionnaires to conduct research. Primary concerns include low response rates, low representativeness, sampling issues, and the comparability of Web-collected data to data collected by other modes [6,44,46,48]. Although these are important concerns that are not fully addressed by our study, we have shown that in a nonrandomized mixed-mode survey for a particularly sensitive health topic, the differences between telephone- and Web-collected responses were minimal in terms of social desirability bias and item nonresponse. This is promising given that Web surveys are significantly cheaper and faster to implement. On the basis of our analysis in this nonrandomized study, we cautiously suggest a comparative strength of Web surveys is that they may provide more truthful results, particularly for questions about sexual behaviors. Although we did see more item nonresponse among the Web group overall, it was mostly nonsignificant and may be addressed through improved Web questionnaire design, such as using prompts that alert respondents if they attempt to move forward without completing a question [2,22].

Our results, although limited by the observational study design, may be important to consider in light of the increasing use of mixed-mode surveys that combine telephone and Web completion modes to reduce costs and increase validity and may help inform future experimental survey methods studies and population-based research in the area of HIV and AIDS.

## Appendix

Multimedia Appendix 1

Multimedia Appendix 2

## Abbreviations

AIDS: acquired immune deficiency syndrome
CI: confidence interval
DK: do not know
HIV: human immunodeficiency virus
OR: odds ratio
SE: standard error
