# Supplementary material for: A Comparison of Web and Telephone Responses From a National HIV and AIDS Survey
Source: JMIR Public Health Surveill. 2016 Jul 29;2(2):e37. doi: 10.2196/publichealth.5184 (PMC4994958; doi:10.2196/publichealth.5184)
Supplement: Multimedia Appendix 1 [file publichealth_v2i2e37_app1.pdf]

Appendix: Full Questions and Response Keys

Questions used in our analysis from the 2011 National HIV/AIDS survey [30]. Note that "don't know/not sure", "prefer not to answer", and skipped questions were all coded as missing.

# in Paper	# in Survey	Question Type	Question and Response Key
1	26	Sensitive	Have you ever been tested for HIV/AIDS for any of the following reasons? (can select multiple responses)<br>o Yes, for insurance purposes<br>o Yes, when donating blood<br>o Yes, for immigration purposes<br>o Yes, to find out if you were infected with HIV<br>o Yes, to participate in a research study<br>o Yes, for another reason (PLEASE SPECIFY) ___________________<br>o No, have never been tested for HIV/AIDS<br>o Don't know/not sure
2	52	Sensitive	*Preamble for next four questions: "The next few questions are strictly for the purposes of understanding sexual behavior. If you are uncomfortable with a particular question, please indicate that you prefer not to answer. Please keep in mind that all of your answers are strictly confidential and that your answers are very important to fully understanding health issues."*<br>Have you ever had sex in your lifetime?<br>o Yes<br>o No<br>o Prefer not to answer
-	53	-	*Note: not used in analysis above but questions 55 and 56 in the questionnaire are nested under this question.*<br>Have you had sexual intercourse in the last 12 months?<br>o Yes<br>o No<br>o Prefer not to answer
3	55	Sensitive	*If Q53=yes*<br>How many different partners have you had sexual intercourse with in the last 12 months?<br>o 1<br>o 2<br>o 3<br>o 4<br>o 5<br>o 6 to 10

			o 11 - 15<br>o 16 – 20<br>o 21 – 30<br>o 31 – 50<br>o More than 50<br>o Prefer not to answer
4	56	Sensitive	*If Q53=yes*<br>Were any of these casual partners? In other words, were they someone that you are not in a regular or long-term relationship?<br>o Yes<br>o No<br>o Prefer not to answer
5	85	Sensitive	What is your annual HOUSEHOLD income from all sources before taxes?<br>o <$20,000<br>o $20,000-$29,999<br>o $30,000-$39,999<br>o $40,000-$49,999<br>o $50,000-$59,999<br>o $60,000-$79,999<br>o $80,000-$99,999<br>o $100,000-$119,999<br>o $120,000-$139,999<br>o $140,000 or more<br>o Prefer not to answer
6	29	Stigma	*Preamble for next two questions: "For each of the following statements, please select an answer using a 7 point scale, where "1" means you completely disagree, "7" means you completely agree and the midpoint "4" means you neither agree nor disagree."*<br>I could not become friends with someone who has HIV/AIDS.<br>o 1- Completely disagree<br>o 2- Disagree<br>o 3- Somewhat disagree<br>o 4- Neither disagree nor agree<br>o 5- Somewhat agree<br>o 6- Agree<br>o 7- Completely agree<br>o 99- Don't know/Not sure
7	30	Stigma	I feel afraid of people living with HIV/AIDS.<br>o 1- Completely disagree<br>o 2- Disagree<br>o 3- Somewhat disagree

			o 4- Neither disagree nor agree<br>o 5- Somewhat agree<br>o 6- Agree<br>o 7- Completely agree<br>o 99- Don’t know/Not sure
8	41	Stigma	People living with HIV/AIDS have the right to be sexually active.<br>o 1- Completely disagree<br>o 2- Disagree<br>o 3- Somewhat disagree<br>o 4- Neither disagree nor agree<br>o 5- Somewhat agree<br>o 6- Agree<br>o 7- Completely agree<br>o 99- Don’t know/Not sure
9	39	Stigma	How comfortable would you be with a close friend or family member dating someone with HIV/AIDS?<br>o 1- Very uncomfortable<br>o 2- Somewhat uncomfortable<br>o 3- Somewhat comfortable<br>o 4- Very comfortable<br>o 9- Don’t know/Not sure
10	37	Stigma	How comfortable or uncomfortable would you be with shopping at a small neighbourhood grocery store, if you found out that the owner had HIV/AIDS?<br>o 1- Very uncomfortable<br>o 2- Somewhat uncomfortable<br>o 3- Somewhat comfortable<br>o 4- Very comfortable<br>o 9- Don’t know/Not sure
11	2	Less-Sensitive	Thinking about different illnesses or diseases, what is the one illness or disease that concerns you the most? (PLEASE WRITE IN ONE RESPONSE IN THE SPACE PROVIDED).<br>o ___________________________________
12	50	Less-Sensitive	In the past year, did you actively seek out or look for information about HIV/AIDS?<br>o Yes<br>o No<br>o Don’t know/Not sure
13	59	Less-Sensitive	Do you recall donating to any charitable or not-for-profit organization in the last year?<br>o Yes

			o No<br>o Don't know/Not sure
14	72	Less-Sensitive	*Preamble: "Please indicate the extent to which you believe each of the following by selecting a number between "1" and "7" where "1" means to no extent at all, "7" means to a very large extent and the midpoint 4 means to a moderate extent."*<br>To what extent do you believe it is government's responsibility to continue to fund HIV/AIDS research?<br>o 1- To no extent at all<br>o 2<br>o 3<br>o 4 - To a moderate extent<br>o 5<br>o 6<br>o 7 - To a very large extent<br>o 99 – Don't know/Not sure
15	8	Less-Sensitive	How knowledge would you say that you are about HIV/AIDS on a scale of 1 to 7, where "1" is not at all knowledgeable and "7" is extremely knowledgeable and the midpoint "4" is moderately knowledgeable?<br>o 1- Not at all knowledgeable<br>o 2<br>o 3<br>o 4 – Moderately knowledgeable<br>o 5<br>o 6<br>o 7 – Extremely knowledgeable<br>o 99 – Don't know/Not sure
