# Supplementary material for: A Comparison of Web and Telephone Responses From a National HIV and AIDS Survey
Source: JMIR Public Health Surveill. 2016 Jul 29;2(2):e37. doi: 10.2196/publichealth.5184 (PMC4994958; doi:10.2196/publichealth.5184)
Supplement: Multimedia Appendix 2 [file publichealth_v2i2e37_app2.pdf]

Supplementary Table 1: Logistic regression analysis of association between mode of questionnaire completion and reporting more than 1 sexual partner in the last 12 months (n=1424).

Variable	Response Category	Socio-demographic adjusted			Socio-demographic and propensity score adjusted		
		Odds Ratio	LLC	ULC	Odds Ratio	LLC	ULC
**Mode of completion**	Telephone	1.00	-	-	1.00	-	-
	Web	3.76	1.86	7.58	3.65	1.80	7.41
**Age**	18-24	1.00	-	-	1.00	-	-
	25-39	0.42	0.25	0.71	0.43	0.25	0.72
	40-49	0.21	0.11	0.40	0.25	0.12	0.52
	50-59	0.17	0.09	0.32	0.20	0.09	0.45
	60+	0.08	0.04	0.19	0.11	0.04	0.31
**Gender**	Male	1.00	-	-	1.00	-	-
	Female	0.49	0.34	0.71	0.50	0.34	0.72
**Education**	≤High school	1.00	-	-	1.00	-	-
	College	1.00	0.57	1.75	0.68	0.24	1.92
	University	0.71	0.40	1.25	0.45	0.14	1.47
	Grad/Post-Grad	0.86	0.44	1.69	0.51	0.13	1.97
**Income**	<$40,000	1.00	-	-	1.00	-	-
	$40,000-$80,000	0.65	0.40	1.05	0.56	0.32	0.99
	>$80,000	0.42	0.25	0.70	0.33	0.15	0.69
	Prefer not to answer	0.28	0.13	0.62	0.26	0.11	0.59
**Region**	West	1.00	-	-	1.00	-	-
	Ontario	1.02	0.65	1.60	1.10	0.68	1.79
	Quebec	1.66	1.02	2.69	1.89	1.08	3.30
	East	0.94	0.45	1.95	0.98	0.47	2.08
	Territories	4.01	0.96	16.86	3.80	0.94	15.30
**Sexual minority**	No	1.00	-	-	1.00	-	-
	Yes	5.70	3.30	9.86	4.97	2.62	9.40
**Propensity score**					7.68	0.06	939.11

LLC=lower limit of 95% confidence interval, ULC= upper limit of 95% confidence interval

Supplementary Table 2: Linear regression of association between mode of questionnaire completion and agreement[a] with statement “I feel afraid of people living with HIV/AIDS” (n=2008).

Variable	Response Category	Socio-demographic adjusted			Socio-demographic and propensity score adjusted		
		β Coefficient	LLC	ULC	β Coefficient	LLC	ULC
**Mode of completion**	Telephone	0.000	-	-	0.000	-	-
	Web	0.000	-0.231	0.231	0.019	-0.214	0.251
**Age**	18-24	0.000	-	-	0.000	-	-
	25-39	-0.018	-0.308	0.272	-0.043	-0.334	0.248
	40-49	-0.067	-0.383	0.250	-0.204	-0.559	0.152
	50-59	-0.168	-0.472	0.137	-0.312	-0.658	0.034
	60+	0.119	-0.186	0.424	-0.123	-0.540	0.293
**Gender**	Male	0.000	-	-	0.000	-	-
	Female	-0.403	-0.562	-0.245	-0.425	-0.586	-0.264
**Education**	≤High school	0.000	-	-	0.000	-	-
	College	-0.173	-0.414	0.068	0.179	-0.293	0.651
	University	-0.264	-0.510	-0.017	0.164	-0.390	0.718
	Grad/Post-Grad	-0.482	-0.755	-0.208	0.013	-0.616	0.641
**Income**	<$40,000	0.000	-	-	0.000	-	-
	$40,000-$80,000	-0.282	-0.515	-0.050	-0.142	-0.430	0.146
	>$80,000	-0.330	-0.561	-0.100	-0.098	-0.458	0.262
	Prefer not to answer	-0.071	-0.349	0.207	0.016	-0.282	0.314
**Region**	West	0.000	-	-	0.000	-	-
	Ontario	0.069	-0.114	0.253	0.000	-0.199	0.199
	Quebec	0.518	0.293	0.743	0.398	0.134	0.661
	East	0.171	-0.165	0.507	0.119	-0.223	0.461
	Territories	0.273	-0.400	0.946	0.340	-0.341	1.021
**Sexual minority**	No	0.000	-	-	0.000	-	-
	Yes	-0.690	-1.009	-0.371	-0.574	-0.912	-0.235
**Propensity score**					-1.567	-3.474	0.341

LLC=lower limit of 95% confidence interval, ULC= upper limit of 95% confidence interval

[a]1-7 Likert scale; 1=completely disagree, 4=neither agree nor disagree, 7=completely agree

Supplementary Table 3: Linear regression of association between mode of questionnaire completion and level of comfort[a] with statement “How comfortable or uncomfortable would you be with shopping at a small neighbourhood grocery store, if you found out that the owner had HIV/AIDS?” (n=1934).

Variable	Response Category	Socio-demographic adjusted			Socio-demographic and propensity score adjusted		
		β Coefficient	LLC	ULC	β Coefficient	LLC	ULC
**Mode of completion**	Telephone	0.00	-	-	0.00	-	-
	Web	0.09	-0.03	0.21	0.07	-0.05	0.19
**Age**	18-24	0.00	-	-	0.00	-	-
	25-39	0.11	-0.05	0.27	0.13	-0.02	0.29
	40-49	0.09	-0.09	0.26	0.21	0.02	0.40
	50-59	-0.09	-0.26	0.08	0.04	-0.15	0.23
	60+	-0.25	-0.41	-0.08	-0.02	-0.24	0.20
**Gender**	Male	0.00	-	-	0.00	-	-
	Female	0.16	0.07	0.24	0.18	0.09	0.26
**Education**	≤High school	0.00	-	-	0.00	-	-
	College	0.18	0.05	0.31	-0.14	-0.41	0.13
	University	0.33	0.20	0.46	-0.06	-0.38	0.26
	Grad/Post-Grad	0.47	0.32	0.62	0.02	-0.35	0.38
**Income**	<$40,000	0.00	-	-	0.00	-	-
	$40,000-$80,000	-0.01	-0.13	0.11	-0.14	-0.28	0.01
	>$80,000	0.03	-0.09	0.15	-0.18	-0.37	0.01
	Prefer not to answer	-0.08	-0.24	0.07	-0.16	-0.33	0.00
**Region**	West	0.00	-	-	0.00	-	-
	Ontario	-0.04	-0.15	0.07	0.02	-0.09	0.14
	Quebec	0.04	-0.07	0.15	0.15	0.02	0.29
	East	0.04	-0.12	0.21	0.09	-0.08	0.26
	Territories	-0.32	-0.69	0.05	-0.38	-0.76	0.01
**Sexual minority**	No	0.00	-	-	0.00	-	-
	Yes	0.19	-0.01	0.39	0.09	-0.13	0.30
**Propensity score**					1.43	0.39	2.47

LLC=lower limit of 95% confidence interval, ULC= upper limit of 95% confidence interval

[a]1-4 Likert scale; 1=very uncomfortable, 4=very comfortable

Supplementary Table 4: Logistic regression analysis of association between mode of questionnaire completion and donation to charity in the last year (n=1996).

Variable	Response Category	Socio-demographic adjusted			Socio-demographic and propensity score adjusted		
		Odds Ratio	LLC	ULC	Odds Ratio	LLC	ULC
**Mode of completion**	Telephone	1.00	-	-	1.00	-	-
	Web	1.61	1.15	2.27	1.63	1.15	2.29
**Age**	18-24	1.00	-	-	1.00	-	-
	25-39	1.34	0.87	2.07	1.33	0.86	2.06
	40-49	2.19	1.34	3.58	2.06	1.14	3.74
	50-59	3.38	2.06	5.56	3.17	1.72	5.84
	60+	6.33	3.83	10.47	5.64	2.58	12.31
**Gender**	Male	1.00	-	-	1.00	-	-
	Female	1.74	1.33	2.28	1.73	1.31	2.27
**Education**	≤High school	1.00	-	-	1.00	-	-
	College	1.29	0.89	1.87	1.47	0.66	3.26
	University	1.75	1.17	2.60	2.05	0.79	5.36
	Grad/Post-Grad	1.70	1.02	2.83	2.05	0.68	6.22
**Income**	<$40,000	1.00	-	-	1.00	-	-
	$40,000-$80,000	2.88	2.03	4.11	3.04	1.93	4.78
	>$80,000	3.05	2.10	4.43	3.33	1.78	6.22
	Prefer not to answer	2.10	1.34	3.29	2.17	1.34	3.52
**Region**	West	1.00	-	-	1.00	-	-
	Ontario	0.83	0.59	1.17	0.81	0.55	1.18
	Quebec	0.49	0.34	0.71	0.47	0.30	0.73
	East	0.58	0.35	0.97	0.57	0.33	0.96
	Territories	1.39	0.17	11.32	1.41	0.18	11.23
**Sexual minority**	No	1.00	-	-	1.00	-	-
	Yes	1.68	0.86	3.27	1.76	0.87	3.56
**Propensity score**					0.54	0.02	13.67

LLC=lower limit of 95% confidence interval, ULC= upper limit of 95% confidence interval

Supplementary Table 5: Linear regression of association between mode of questionnaire completion and self-perceived HIV knowledge (n=2022).

Variable	Response Category	Socio-demographic adjusted			Socio-demographic and propensity score adjusted		
		β Coefficient	LLC	ULC	β Coefficient	LLC	ULC
**Mode of completion**	Telephone	0.00	-	-	0.00	-	-
	Web	-0.06	-0.23	0.11	-0.05	-0.22	0.12
**Age**	18-24	0.00	-	-	0.00	-	-
	25-39	0.13	-0.09	0.34	0.11	-0.10	0.33
	40-49	-0.04	-0.27	0.20	-0.09	-0.35	0.16
	50-59	-0.04	-0.28	0.19	-0.11	-0.36	0.15
	60+	-0.30	-0.52	-0.07	-0.40	-0.69	-0.11
**Gender**	Male	0.00	-	-	0.00	-	-
	Female	0.12	0.00	0.23	0.11	-0.01	0.22
**Education**	≤High school	0.00	-	-	0.00	-	-
	College	0.27	0.10	0.45	0.42	0.08	0.77
	University	0.42	0.24	0.60	0.60	0.19	1.01
	Grad/Post-Grad	0.58	0.37	0.79	0.79	0.32	1.26
**Income**	<$40,000	0.00	-	-	0.00	-	-
	$40,000-$80,000	0.11	-0.06	0.27	0.17	-0.04	0.37
	>$80,000	0.13	-0.04	0.29	0.22	-0.03	0.48
	Prefer not to answer	0.01	-0.20	0.22	0.04	-0.18	0.27
**Region**	West	0.00	-	-	0.00	-	-
	Ontario	0.13	-0.01	0.26	0.10	-0.05	0.25
	Quebec	0.51	0.35	0.66	0.46	0.27	0.64
	East	-0.16	-0.39	0.07	-0.18	-0.42	0.05
	Territories	-0.32	-0.75	0.10	-0.29	-0.72	0.13
**Sexual minority**	No	0.00	-	-	0.00	-	-
	Yes	0.84	0.58	1.11	0.89	0.61	1.17
**Propensity score**					-0.66	-2.01	0.68

LLC=lower limit of 95% confidence interval, ULC= upper limit of 95% confidence interval
[a]1-7 Likert scale; 1=not at all knowledgeable, 4=moderately knowledgeable, 7=extremely knowledgeable

Supplementary Table 6: Logistic regression analysis of association between mode of questionnaire completion and missing/DK response for HIV/AIDS testing (n=2027).

Variable	Response Category	Socio-demographic adjusted			Socio-demographic and propensity score adjusted		
		Odds Ratio	LLC	ULC	Odds Ratio	LLC	ULC
**Mode of completion**	Telephone	1.00	-	-	1.00	-	-
	Web	8.68	2.63	28.65	8.04	2.46	26.29
**Age**	18-24	1.00	-	-	1.00	-	-
	25-39	1.17	0.36	3.79	1.27	0.38	4.22
	40-49	2.40	0.73	7.88	3.59	0.82	15.76
	50-59	3.39	1.10	10.49	5.15	1.28	20.71
	60+	2.38	0.78	7.29	4.68	0.90	24.30
**Gender**	Male	1.00	-	-	1.00	-	-
	Female	0.91	0.58	1.44	0.97	0.60	1.57
**Education**	≤High school	1.00	-	-	1.00	-	-
	College	0.71	0.36	1.39	0.27	0.06	1.28
	University	0.73	0.37	1.47	0.22	0.03	1.55
	Grad/Post-Grad	0.59	0.26	1.31	0.15	0.02	1.29
**Income**	<$40,000	1.00	-	-	1.00	-	-
	$40,000-$80,000	1.08	0.54	2.17	0.71	0.28	1.83
	>$80,000	1.05	0.52	2.14	0.51	0.14	1.88
	Prefer not to answer	1.36	0.63	2.94	1.04	0.44	2.43
**Region**	West	1.00	-	-	1.00	-	-
	Ontario	1.04	0.61	1.77	1.27	0.70	2.32
	Quebec	0.48	0.22	1.06	0.69	0.27	1.78
	East	1.78	0.82	3.83	2.06	0.94	4.49
	Territories	1.47	0.31	6.95	1.16	0.26	5.20
**Sexual minority**	No	1.00	-	-	1.00	-	-
	Yes	0.25	0.03	1.79	0.17	0.03	1.16
**Propensity score**					84.81	0.12	60526

LLC=lower limit of 95% confidence interval, ULC= upper limit of 95% confidence interval

Supplementary Table 7: Logistic regression analysis of association between mode of questionnaire completion and missing/DK response for comfort with shopping at small grocery store owned by someone who has HIV/AIDS (n=2027).

Variable	Response Category	Socio-demographic adjusted			Socio-demographic and propensity score adjusted		
		Odds Ratio	LLC	ULC	Odds Ratio	LLC	ULC
**Mode of completion**	Telephone	1.00	-	-	1.00	-	-
	Web	2.99	1.41	6.33	3.12	1.47	6.63
**Age**	18-24	1.00	-	-	1.00	-	-
	25-39	0.94	0.42	2.14	0.90	0.39	2.03
	40-49	0.70	0.26	1.86	0.55	0.20	1.57
	50-59	1.53	0.66	3.56	1.20	0.46	3.14
	60+	1.27	0.54	2.97	0.84	0.26	2.76
**Gender**	Male	1.00	-	-	1.00	-	-
	Female	0.84	0.54	1.31	0.81	0.52	1.27
**Education**	≤High school	1.00	-	-	1.00	-	-
	College	0.54	0.30	1.00	0.94	0.25	3.59
	University	0.54	0.29	0.99	1.04	0.22	4.97
	Grad/Post-Grad	0.31	0.13	0.70	0.67	0.10	4.31
**Income**	<$40,000	1.00	-	-	1.00	-	-
	$40,000-$80,000	0.61	0.32	1.17	0.77	0.34	1.73
	>$80,000	0.86	0.46	1.62	1.26	0.46	3.44
	Prefer not to answer	2.16	1.13	4.13	2.50	1.23	5.08
**Region**	West	1.00	-	-	1.00	-	-
	Ontario	0.68	0.41	1.14	0.61	0.34	1.09
	Quebec	0.93	0.53	1.64	0.75	0.34	1.67
	East	0.26	0.06	1.11	0.24	0.06	1.04
	Territories	1.39	0.33	5.96	1.56	0.37	6.65
**Sexual minority**	No	1.00	-	-	1.00	-	-
	Yes	0.20	0.03	1.51	0.24	0.03	1.83
**Propensity score**					0.09	0.00	12.78

LLC=lower limit of 95% confidence interval, ULC= upper limit of 95% confidence interval

Supplementary Table 8: Logistic regression analysis of association between mode of questionnaire completion and missing/DK response for most concerning illness or disease (n=2027).

Variable	Response Category	Socio-demographic adjusted			Socio-demographic and propensity score adjusted		
		Odds Ratio	LLC	ULC	Odds Ratio	LLC	ULC
**Mode of completion**	Telephone	1.00	-	-	1.00	-	-
	Web	3.01	1.68	5.38	3.02	1.67	5.47
**Age**	18-24	1.00	-	-	1.00	-	-
	25-39	1.45	0.83	2.52	1.44	0.83	2.51
	40-49	1.10	0.58	2.08	1.07	0.53	2.15
	50-59	1.07	0.59	1.96	1.04	0.53	2.04
	60+	1.11	0.62	1.99	1.06	0.47	2.37
**Gender**	Male	1.00	-	-	1.00	-	-
	Female	0.68	0.50	0.93	0.68	0.50	0.93
**Education**	≤High school	1.00	-	-	1.00	-	-
	College	0.88	0.56	1.39	0.95	0.37	2.46
	University	0.84	0.52	1.35	0.91	0.29	2.87
	Grad/Post-Grad	0.68	0.38	1.21	0.75	0.20	2.86
**Income**	<$40,000	1.00	-	-	1.00	-	-
	$40,000-$80,000	0.88	0.57	1.36	0.91	0.54	1.53
	>$80,000	0.76	0.49	1.18	0.79	0.40	1.59
	Prefer not to answer	1.62	1.00	2.62	1.64	0.98	2.76
**Region**	West	1.00	-	-	1.00	-	-
	Ontario	0.78	0.54	1.12	0.77	0.51	1.15
	Quebec	0.90	0.59	1.36	0.87	0.53	1.45
	East	0.81	0.42	1.54	0.80	0.41	1.53
	Territories	0.69	0.15	3.10	0.70	0.16	3.07
**Sexual minority**	No	1.00	-	-	1.00	-	-
	Yes	1.19	0.63	2.25	1.22	0.63	2.36
**Propensity score**					0.72	0.01	37.30

LLC=lower limit of 95% confidence interval, ULC= upper limit of 95% confidence interval
